# Endometrium and Ovarian cancer synchronous to endometriosis –A retrospective study of our experience of 7 years– 

**Published:** 2010-02-25

**Authors:** I Grammatikakis, S Zervoudis, N Evangelinakis, V Tziortzioti

**Affiliations:** Lito Maternity Hospital, AthensGreece

**Keywords:** ovarian endometriosis, synchronous genital cancers

## Abstract

Purpose: Although endometriosis is a benign disorder, recent studies suggest that endometriosis could be viewed as a neoplastic process. The objective of this study is to explore the epidemiology of synchronous neoplasms (SPN) in women with severe endometriosis.

Patients and Methods: The prevalence of SPN in cases with endometriotic ovarian cysts that underwent surgery at ‘Lito’ Maternity Hospital of Athens and at Anticancer Institute of Bucharest was investigated. The review period was from the year 2000 to 2008. The medical records and pathology were reviewed to confirm the diagnosis and stage of tumors.

Results: Five women with synchronous cancer of the genital tract were identified. All of our patients had a grade–Ⅰ endometrioid carcinoma of the uterus (Ⅰa in 3, Ⅱb in 2). Myometrium was invaded in less than 1/3, in 4 cases, and less than ½ in one case. Similarly, 4 out of 5 ovarian cancers were endometrioid, while one was serum cystadenosarcoma. All of the ovarian malignancies were grade Ⅰ (Ⅰb in 3 and Ⅰa in 2). The median diameter of the ovarian neoplasias was of 4.3cm, as opposed to 4.5cm that was the median diameter of all endometrioid cysts. When the larger ovarian malignant cyst in each patient was accounted, the median diameter was calculated as having 5.8cm.

Conclusions: Women with synchronous primary cancers of the endometrium and ovary have distinct clinical characteristics including younger age, premenopausal status, and nulliparity. This suggests that a hormonal ‘field effect’ may account for the development of these simultaneous endometrioid cancers, supporting the theory of estrogen receptors.

## Introduction

Synchronous primary neoplasms (SPN) of the female reproductive tract represent a rare situation with a special clinical interest. It is an idiomorphous phenomenon with unknown pathogenesis and etiology. It occurs in 10% of all women with ovarian cancer and 5% of all women with endometrial cancer [1]. A first effort to delineate a set of pathologic criteria to distinguish the metastatic disease from synchronous primary tumors was made by Ulbright and Roth [2], in 1985. Nowadays, a similar but more extensive list of clinicopathologic features created by Scully et al. [3] is used to differentiate between endometrial cancer with metastasis to the ovary, ovarian cancer with metastasis to the endometrium, and independent primary cancers (Table 1).

**Table 1:** Endometrioid tumors of the ovary and endometrium, Independent primary tumors. [3]

Independent primary tumors
Histological dissimilarity of the tumors
No or only superficial myometrial invasion of endometrial tumor
No vascular space invasion of endometrial tumor
Absence of other evidence of spread of endometrial tumor
Atypical endometrial hyperplasia additionally present
Ovarian unilateral tumor (80–90%) of cases
Ovarian tumor located in parenchyma
No vascular space invasion, surface implants, or predominant hilar location in ovary
Absence of other evidence of spread of ovarian tumor
Ovarian endometriosis present
Different ploidy of DNA indices, if aneuploid, of the tumors
Dissimilar molecular genetic or karyotypic abnormalities in the tumors

In our institution, gynecologic pathologists use these criteria to determine whether the tumors of the endometrium and ovary represent a metastatic disease or independent primary tumors. The most common hypothesis about pathogenesis is that embryologically, similar tissues may develop synchronous neoplasms, when subjected to hormonal influences or to carcinogens. Primary malignancies of the ovary and endometrium have been investigated in many trials. In addition, endometriosis may be the precursor of clear cell or endometrioid ovarian cancer. The pathological and epidemiological evidence demonstrates a strong association with ovarian cancer as well. Estrogen receptors may be responsible for the development of multiple primary malignancies in predisposed tissue. Actually, the presence of endometriosis is associated with an increased risk of synchronous primary neoplasms mainly in ovary and endometrium. In our study, we present the frequency and types of synchronous cancers of female genital tract that appear in women surgically treated for endometriosis.

## Patients and methods

In the present study, we investigated the prevalence of SPN in cases with endometriotic ovarian cysts that underwent surgery at ‘Lito’ Maternity hospital of Athens and at Anticancer Institute of Bucharest. In our analysis, a total number of 811 women have been included, after clinical and ultrasound diagnosis of ovarian endometriomas (transvaginal ultrasound). All the patients underwent surgery and some of them underwent hysterectomy. The review period was from the year 2000 to 2008. The medical records and pathology were reviewed to confirm the diagnosis and stage of tumors. Stage was assigned according to the FIGO criteria. Pathology specimens of SPN were reviewed to confirm original pathologic type and grade. The tumor grades for ovarian and endometrial cancer were assigned as it follows: grade Ⅰ (well differentiated), grade Ⅱ (moderately differentiated), grade Ⅲ (poorly differentiated).

## Results

During the reported period of time, 811 patients underwent surgery for the excision of ovarian endometriosis (severe endometriosis). The mean age of the women was 40.9 years old (19–68 years old). In 380 (46.8%) cases the mass was located at the left adnexal, in 294 (36.2%) in the right and in 137 (17%) women in both ovaries. Diameter of masses ranged from 2–15 cm, the median diameter being of 4.95 cm. 

Based on the pathologic reports, nine women with synchronous cancers of the genital tract were identified. After reviewing, though, the pathological sections 4 out of 9 patients were identified as metastatic cases. So, five (0.62%) patients were identified as having synchronous primary neoplasms. The mean age of the five women was of 54.2 years old. Three patients were nulliparous. All of our patients had a grade–Ⅰ endometrioid carcinoma of the uterus, while stage was Ⅰa in 3 and Ⅰb in 2. The myometrium was invaded less than 1/3 in 4 cases and less than 2/3 in all cases. In the case where carcinoma invaded more than 1/3 of the myometrium, simple atypical hyperplasia of the endometrium was also identified. Similarly, 4 out of 5 ovarian cancers were endometrioid, while one was serous cystadenosarcoma. All of the ovarian malignancies were grade Ⅰ (three of them were staged with ࡰb and Ⅱ as Ⅰa). In two cases, ovarian malignancy was positive for HER–2/neu. The median diameter of the ovarian neoplasias was of 4.3cm, compared to 4.5cm that was the median diameter of all endometrioid cysts. When the larger ovarian malignant cyst in each patient was accounted, the median diameter was calculated as having 5.8cm. In two cases, ovarian malignancy was detected only to the right ovary, while to the rest it was bilateral. What is interesting is that in 2 out of 3 cases of bilateral ovarian malignancies the larger cyst was in the right ovary (Table 2).

**Table 2:** Histopathological analysis of the 5 patients with synchronous primary carcinomas of the endometrium and ovary. EN: Endometrioid, CS: Serum cystedenosarcoma, R: Right, B: Bilateral. *Simple atypical hyperplasia was also identified

Case no	Histologic type and grade	Histologic type and grade	Staging (FIGO)	Staging (FIGO)	Myometrial invasion	Involved ovaries	Endometriosis
	Endo–metrium	Ovary	Endo–metrium			
1	EN –Ⅰ	EN	Ⅰa	Ⅰb	<1/3	R	+
2	EN –Ⅰ	CS	Ⅰb	Ⅰb	<1/3	B	+
3	EN –Ⅰ *	EN	Ⅰa	Ⅰa	<1/2	R	+
4	EN –Ⅰ	EN	Ⅰa	Ⅰa	<1/3	B	+
5	EN –Ⅰ	EN	Ⅰb	Ⅰa	<1/3	B	+

## Discussion

The synchronous occurrence of endometrial and ovarian cancer is well known but still poses a diagnostic dilemma. A still existing diagnostic dilemma is the synchronous occurrence of malignancies of the female genital tract. In cases of different histologic types, the dilemma of which cancer is primary and which is metastatic remains. On the other hand, if the histologic types are different it is easy to identify the coexistence of the two primary cancers. Although immunohistochemical and DNA flow cytometric studies have been used to distinguish between neoplasms of similar histology, the differential diagnosis still lies primarily on conventional clinicopathologic criteria. It is important to separate women with two primary cancers from those with metastases, since prognosis is significantly better for the first group. 

The most frequently documented synchronous malignancies are those of the ovary and endometrium. It is well known the fact that the simultaneous malignancies are predominantly of low stage and this finding was confirmed by our study as well. Earlier studies suggested that women diagnosed with synchronous primary cancers have a better overall prognosis than if their disease was classified as single organ disease with metastasis [4–6]. A study undergone by the Gynecologic Oncology Group (GOG) on 74 patients with synchronous cancers of the genital tract, reported a 5–year survival of 86% and a 10– year survival of 80% [1]. Few studies, however, focused on risk factors in these patients.

Several hypotheses have been proposed regarding the simultaneous involvement of the endometrium and ovary. The most popular hypothesis concerning the pathogenesis of this extremely rare condition is that estrogen receptors may be responsible for the development of multiple primary malignancies in predisposed tissues. Another hypothesis is that an ‘extended’ or secondary mullerian system exists, so that the similarity of the female's upper genital tracts undergoing common metaplastic diseases could be explained. Endometriosis seems to be a precursor of a clear cell or endometrioid ovarian cancer. The histopathology and epidemiological evidence demonstrates a strong association between endometriosis and ovarian cancer. 

This is one of the few studies that, instead of focusing on women with synchronous neoplasms of the genital tract and identifying the percentage of endometriosis on them, goes the other way round; in a population with moderate and severe endometriosis we identified the prevalence of synchronous cancers. In our cohort, median age at diagnosis was of 54 years old in women with synchronous primary cancers of the endometrium and ovary. In contrast, women who develop endometrial or ovarian cancer alone are predominantly postmenopausal and in the sixth or seventh decade of life. Previous studies have also reported a younger median age in patients with synchronous primary endometrial and ovarian cancers. Eifel et al. [4] (n=29), in particular, found a younger than expected median age of 41 years old in women with endometrioid/endometrioid cancers, while GOG (n=74) reported a median age of 49 years old [1]. Herrinton et al. [7] reported that 39% of patients were under the age of 50 at the time of diagnosis in a case–control study (n=56). Finally, in studies examining young women with endometrial cancer (age less than 45 years), 10–29% of these young women were found with synchronous ovarian cancers [8,9]. 

Our study indicates that women with synchronous primary cancers of the endometrium and ovary have distinct clinical characteristics including younger age, premenopausal status, and nulliparity. This suggests that a hormonal ‘field effect’ may account for the development of these simultaneous endometrioid cancers, supporting the theory of estrogen receptors. Eifel et al. [4] also suggested that the response of the uterine corpus, fallopian tubes, and ovarian epithelium as a morphologic unit could explain the development of synchronous endometrioid tumors in different components of the mullerian system. Future studies are needed to further evaluate the role of estrogen in these synchronous endometrioid cancers of the endometrium and ovary.
